# Megavolt bremsstrahlung measurements from linear induction accelerators demonstrate possible use as a FLASH radiotherapy source to reduce acute toxicity

**DOI:** 10.1038/s41598-021-95807-9

**Published:** 2021-08-24

**Authors:** Stephen E. Sampayan, Kristin C. Sampayan, George J. Caporaso, Yu-Jiuan Chen, Steve Falabella, Steven A. Hawkins, Jason Hearn, James A. Watson, Jan-Mark Zentler

**Affiliations:** 1 Lawrence Livermore National Laboratory, P.O. Box 808, Livermore, CA 94551 USA; 2 Opcondys, Inc., 600 Commerce Court, Manteca, CA 95336 USA; 3 Department of Radiation Oncology, University of Michigan, Ann Arbor, MI 48109 USA

**Keywords:** Electrical and electronic engineering, Radiotherapy, Health care, Medical research, Oncology, Physics

## Abstract

Recent studies indicate better efficacy and healthy tissue sparing with high dose-rate FLASH radiotherapy (FLASH-RT) cancer treatment. This technique delivers a prompt high radiation dose rather than fractional doses over time. While some suggest thresholds of > 40 Gy s^−1^ with a maximal effect at > 100 Gy s^−1^, accumulated evidence shows that instantaneous dose-rate and irradiation time are critical. Mechanisms are still debated, but toxicity is minimized while inducing apoptosis in malignant tissue. Delivery technologies to date show that a capability gap exists with clinic scale, broad area, deep penetrating, high dose rate systems. Based on these trends, if FLASH-RT is adopted, it may become a dominant approach except in the least technologically advanced countries. The linear induction accelerator (LIA) developed for high instantaneous and high average dose-rate, species independent charged particle acceleration, has yet to be considered for this application. We review the status of LIA technology, explore the physics of bremsstrahlung-converter-target interactions and our work on stabilizing the electron beam. While the gradient of the LIA is low, we present our preliminary work to improve the gradient by an order of magnitude, presenting a point design for a multibeam FLASH-RT system using a single accelerator for application to conformal FLASH-RT.

## Introduction

Recent studies indicate better efficacy with high dose-rate FLASH radiotherapy treatment (FLASH-RT). This technique delivers a prompt high radiation dose, sometimes in a single treatment rather than spacing the dose over a longer period of time^1–3^. Review articles abound (see for instance^4–8^). Some suggested that the healthy tissue sparing effect associated with FLASH-RT has a threshold of 40 Gy s^−1^, with a maximal effect occurring at > 100 Gy s^−1^. Highest instantaneous in vivo or in vitro dose rates reported were ≥ 10^7^ Gy s^−1^ in a single 23-MeV, 1 ns, proton pulse or 7 MeV electrons^9,10^. Photon instantaneous dose rates were typically below 10^4^ Gy s^−1^ (Table 1). These surveys hypothesized mechanisms that include oxygen depletion or a modified immune response. Very recent in vivo, in vitro, and water phantom studies and TRAX-CHEM Monte Carlo simulations, however, yielded a consistent conclusion: oxygen partial pressure depletion alone (measured to be 1–3 mmHg) was not a suitable or sufficient mechanism to explain the FLASH effect^11–13^. Thus, alternate mechanisms need exploration.

**Table 1:** Present methods of FLASH-RT delivery.

Desig.	System	Technology		Energy, MeV	Instantaneous dose rate (max.) Gy s^−1^	Average dose rate, Gy s^−1^	Nominal area, cm^2^	References
		Type system	Scale					
**Electrons**								
a	Oriatron (eRT6)	RF Linac	Clinical	4.9–6	≈ 10^7^	1030–1080	55.0	^17^
b	Kinetron	RF Linac	Clinical	4–5	2 × 10^9^	750	3.10	^1^
c	ELBEc	Superconducting RF Linac	Large laboratory—Helmholtz-Zentrum Dresden-Rossendorf	5–40	5 × 10^10^	0.86–4.5 × 10^6^	0.79	^19^
d	Elekta Precise	RF Linac	Clinical	≈ 8	> 10^3^	300	19.6	^20^
e	Varian Clinac 21EX	RF Linac	Clinical	9, 20	6.1 × 10^3^	35–210	12.6	^18^
**Photons**								
f	ESRF (ID17)	Synchrotron	Large laboratory—European synchrotron radiation facility	0.105	8 × 10^3^	14,000	0.50	^21^
g	IMBL	Synchrotron	Large laboratory—imaging and medical beam-line, Australian synchrotron	0.94	–	4441	0.60	^22^
h	MXR 160/22	X-ray tube	Clinical	0.16	≈ 10^2^	114	0.79	^23^
**Protons**								
i	Proteus IBA	Superconducting synchro-cyclotron	Specialized clinic	230	3.9 × 10^3^	39.1	0.79	^24,25^
j	HyperScan Mevion	Superconducting synchro-cyclotron	Specialized clinic	230	1.7 × 10^4^	130	1.10	^8,26^
k	ProBeam Varian (based on publications)	Superconducting isochronous cyclotron	Specialized clinic	250	–	40.0	3.00	^8,27^
l	SNAKE Experimental (micro-beam)	Tandem Van de Graaff accelerator using a beam buncher	Large laboratory—Maier-Leibnitz-Laboratorium	20	7 × 10^7^	8.0 × 10^5^	7.5 × 10^–5^	^28^

As pointed out by Vozenin et al., some data appears under-reported, potentially leading to uncertainty in the overall conclusions; better standardization in reporting critical parameters is required for the field to advance^14^. For instance, geometric parameters such as irradiated volume as well as beam structure, pulse dose and width, number of pulses, and duty cycle allow thorough interpretation of results. More specifically, direct experimental evidence points to instantaneous dose rate (e.g., dose within a pulse) and time as critical parameters.

In earlier studies, irradiation of human lymphocytes with 7 MeV electrons showed cell sparing effects when the dose rate increased (from ≈ 10^6^ to 10^8^ Gy s^−1^ for 2 μs to 25 ns pulse widths, respectively) while maintaining constant total dose^10^. More recent evidence showed reproducibility of the FLASH effect at an instantaneous dose-rate > 1.8 × 10^5^ Gy s^−1^ with an overall irradiation time ≈ 200 ms^15^.

**Figure 1:**
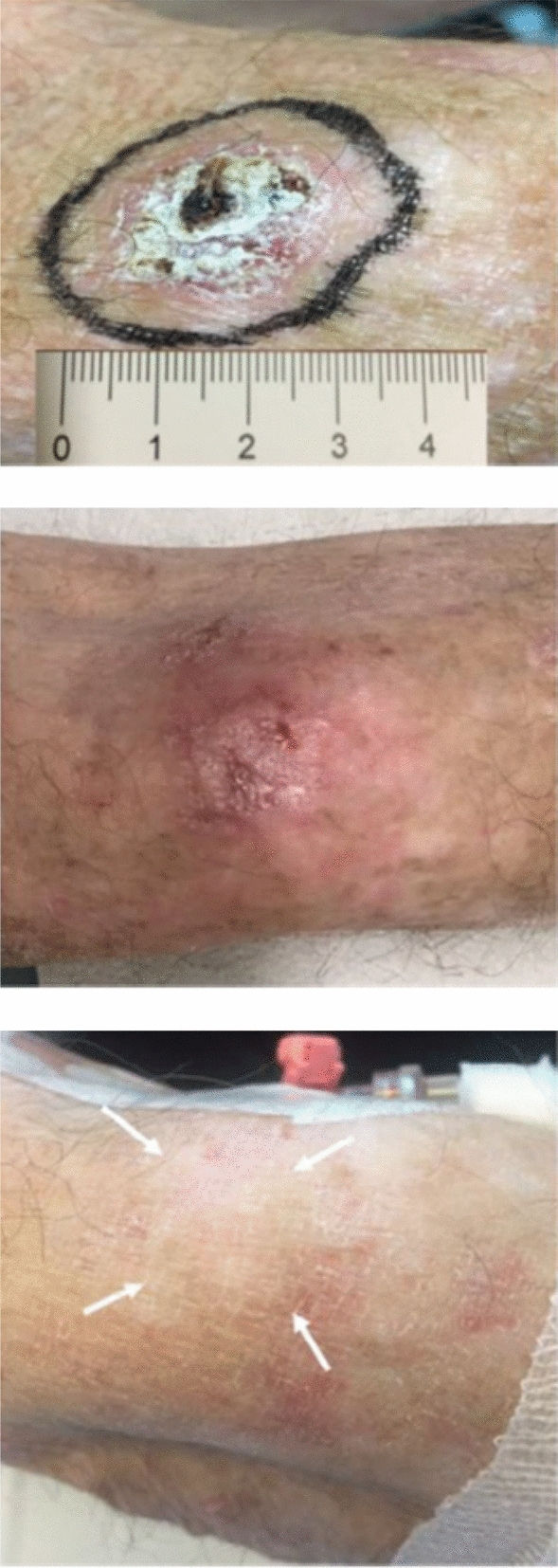
Effects of 167 GY/s (15-Gy in 90-ms) electron beam treatment of a 3.5-cm skin tumor in the first FLASH-RT of a human subject. Top: Day 0, middle: Day 21, bottom: ~ Day 150^16^ (reprinted with permission, License No. 5054930939964).

Human trials are still very limited with one report of 167 Gy s^−1^ (15-Gy in 90-ms) delivered for treatment of a 3.5-cm diameter skin tumor (Fig. 1). This treatment used electrons from an Oriatron eRT6 5.6 MeV microwave LINAC at the Lausanne University Hospital^16,17^. Although it was not possible to draw any firm conclusions, the trial demonstrated treatment was feasible and safe and showed a favorable outcome for normal skin. Tumor response appeared rapid and complete.

## FLASH-RT delivery

### Conventional medical accelerators

**Figure 2:**
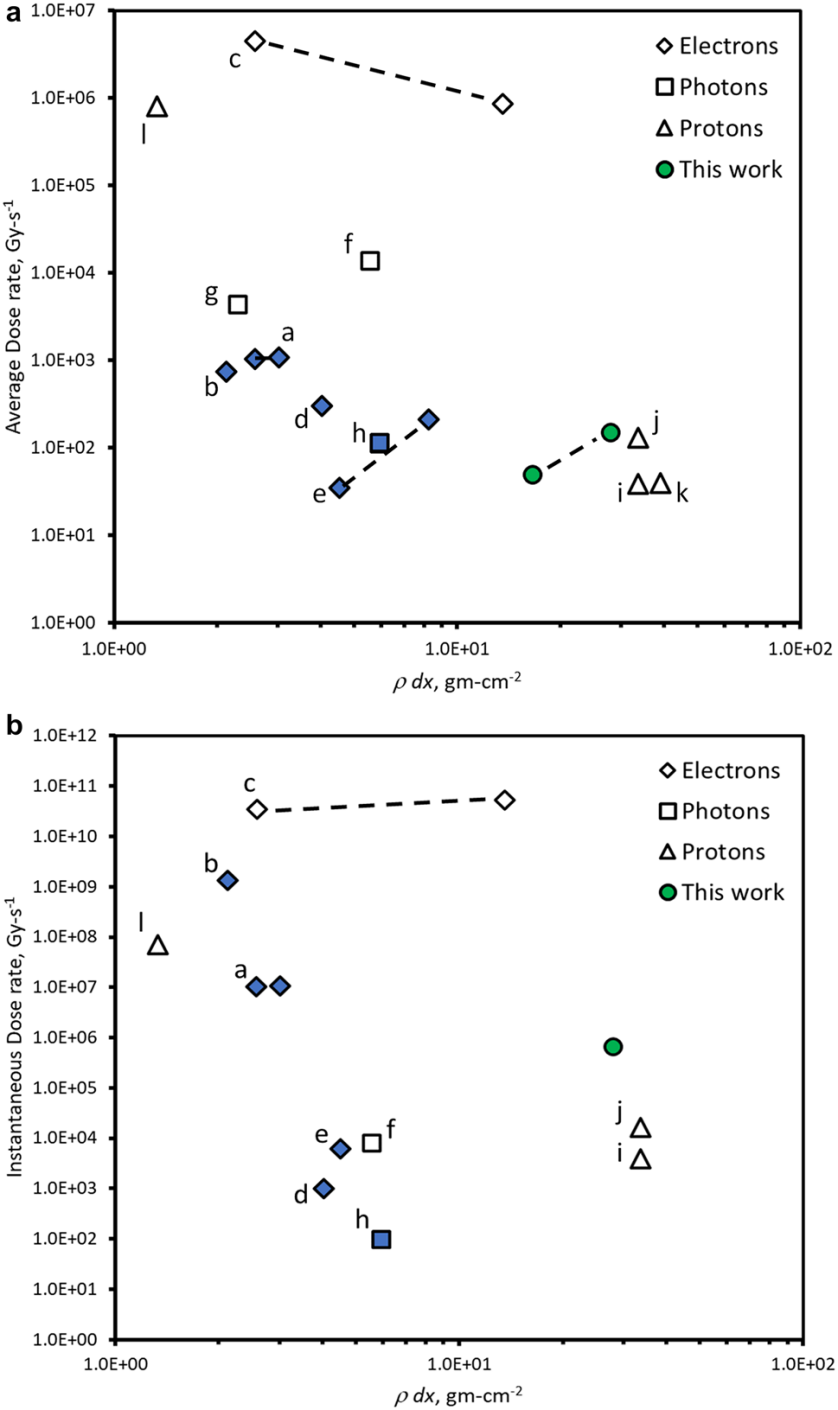
(**a**) A system survey of FLASH-RT average dose rate and (**b**) instantaneous dose rate as a function of range calculated from details obtained from the literature. See Table 1 for specific details. Solid blue markers designate clinic scale systems that can be placed in an approximately 100 m^3^ radiation vault. Dotted line signifies the approximate range of a particular single system. Definitions are based on Wilson and Esplen with calculations as described in “Methods” section^5,6^.

A survey of FLASH-RT delivery systems is shown in Fig. 2 with added details provided in Table 1. We show the need for clinic scale multi-MV FLASH-RT systems. Here we plot the dose rate and instantaneous dose rate as a function of range (in terms of *R* = *ρ dx*) for electrons, photons, and protons^1,6,8,17–28^. We make a distinction between clinic scale systems that would fit in a nominal treatment vault (≈ 100 m^3^, solid blue filled markers) and more costly research centers as well as specialized high energy proton facilities (open markers). Each of these facilities provides a nominal FLASH-RT area ranging from 7.5 × 10^–5^ cm^2^ at a range of about 1.3 g cm^−2^ for 20 MeV protons to the largest area of 55 cm^2^ at a range of about 3 g cm^−2^ for 6 MeV electrons. Energetic proton treatment areas ranged from 0.8 to 3 cm^2^ corresponding to a range of 30 to 40 g cm^−2^ for 230 MeV and above.

In a specific study, mice were irradiated with microbeam radiation therapy (MRT) using a synchrotron 95 keV source^29^. The MRT beam was made up of quasi-parallel micro-planar beams with a width of 25 to 100 µm that were spaced from 100 to 400 µm (i.e., valleys). Dose rates in the beams were between 276 to 319 Gy s^−1^; scatter resulted in an order of magnitude lower dose rate (presumed to be ≈ 30 Gy s^−1^) and total dose in the valleys. Toxicity post irradiation was quantified by the TD_50_ metric based on > 15–20% weight loss, severe diarrhea, and moribund behavior. What was striking about the conclusion was that the MRT FLASH valley total dose (between 3.8 and 7.2 Gy at TD_50_ depending on irradiation site) was a better predictor of acute toxicity, having roughly the same effects as total dose using conventional (0.05–0.06 Gy s^−1^ at 93 keV) and synchrotron (37–41 Gy s^−1^ at 124 keV) delivery. Further, the total dose for TD_50_ at the highest delivery rates increased as the treatment volume was reduced and were between 116 and 261 Gy greater. These results suggest that for FLASH-RT to be healthy tissue sparing, both the periphery and exit edge of the radiation needs to be above the threshold required for the effect; toxicity can be induced by low FLASH-RT dose rates. Further it also suggests that the volume of low dose rate should be minimized.

To increase the treatment area, beam scanning has been proposed. But we were unable to clearly delineate between collimated and hard-edge beams so as to minimize the delivery volume below the FLASH-RT threshold. For a typical Gaussian shaped beam resulting from a non-zero emittance, the dose rate is significantly lower at the beam edges which may result in a reduced tissue sparing FLASH effect^5,29^. Thus, it is not clear if there are biological consequences of irradiating with FLASH contributions below 100% as the approach has only started to be explored; it is unknown how this approach will influence the overall effect^30^.

At present, clinic scale machines for FLASH-RT (mainly electrons) are limited to shallow tumors while deep seated tumors require highly specialized and costly proton systems. Protons have the advantage of being more conformal to the tumor volume. But as of 2017, only 1% of the world cancer patients are treated with protons or heavy particles ($30–100 M US)^31^.

While many of the clinic scale FLASH-RT electron systems can be converted to MV photons, beam loading and fundamental beam stability limitations set practical limits to below ampere level beams and low instantaneous and mean dose rates for single accelerator systems^32^.

Clinic scale electron accelerators rely on the longitudinal resonance of a sinusoidally varying electromagnetic field in parallel with the electron motion; electrons in resonance experience a net positive accelerating gradient (i.e., the energy gained by a charge particle per length of accelerator)^33^. Increased dose rates require higher current. Higher currents, however, affect the electromagnetic wave resonance with the electron longitudinal motion and can result in a lower gradient^34,35^. For instance, an increase from 0.3-A peak current to 1.2-A peak current reduced the exit gradient from 12.7 to 2.5-MV m^−1^ resulting in lower output energy^36^. Further, the beam break-up instability in these systems, which induces uncontrolled transverse beam motion that causes the beam to be lost to the walls, scales as the square-root of the beam current divided by the radius of the cavity^37,38^. This effect can shorten the beam pulse at higher currents.

### Linear induction pulsed power accelerator technology

Pulsed power accelerators store energy over a comparatively long time then discharge in 10 s to 100 s of nanoseconds to deliver pulsed high-power beams. While anecdotal, one of the authors is aware of three separate accounts of the same accidental FLASH dose event that occurred in the mid-1960s. A single pulse, 7–8 MeV, 50 ns pulsed power FLASH electron accelerator accidentally fired while an individual was in the radiation vault. At least one account purports the individual observed a “blue flash” consistent with the recent single human trial^16^. The individual received near the LD_50_ bremsstrahlung dose with little, if any, clinical effect^39^. Soon afterwards, this same type of pulsed power accelerator was used in some of the original FLASH radiation effect studies on mammalian tissue. That research served as the basis for the work today^40^.

The linear induction accelerator (LIA) is based on a pulsed power approach and is mature^41^. It was developed in the 1960s to overcome the beam current limitations in microwave linear accelerators. The systems are inherently high instantaneous and high dose rate FLASH systems. When converted to bremsstrahlung, systems have demonstrated the equivalent instantaneous dose rates > 10^7^ Gy s^−1^.

**Figure 3:**
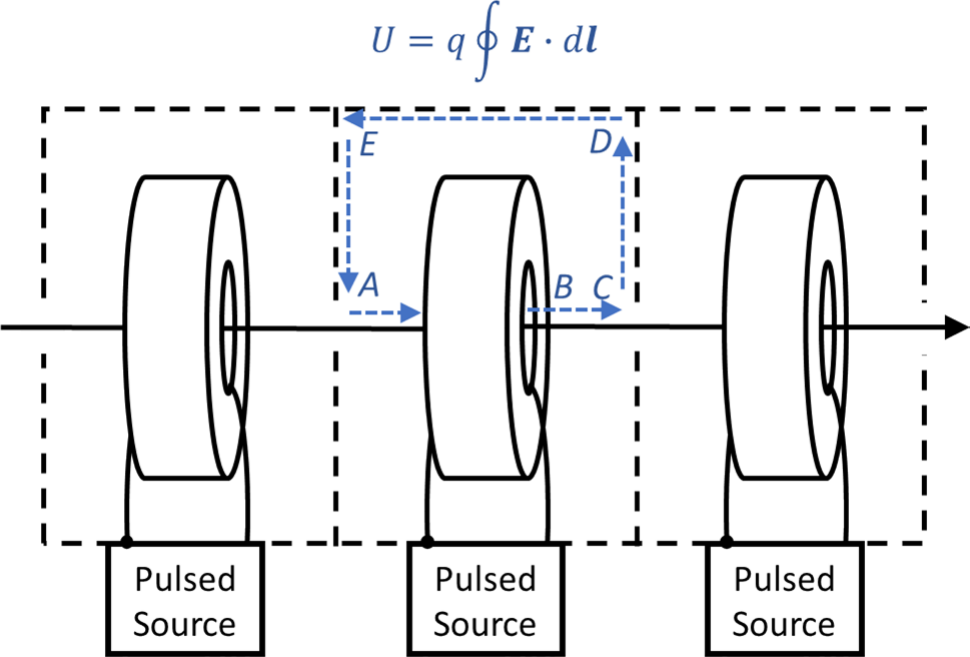
Acceleration of charged particles (on axis arrow) with a linear induction accelerator (LIA). Here *U* is the energy gain, *q* is the particle charge, and ***E*** is the electric field. Magnetic cores are depicted by the toroids and the dotted lines indicate conductive surfaces. One side of the pulsed source is referenced to the accelerator cavity interior (represented by the dot). The electric field outside the structure is everywhere essentially zero within the diffusion time scales of the conductive walls. Thus, the line integral is zero from segments C to A as the electric field is shorted by the conducting wall. The contribution along AB is also zero as the pulsed source output is referenced to the cavity wall. The only contribution is along line segment BC, where charge particles gain energy during the pulse. *N* cavities are arranged in series to provide a total acceleration of *NV*_*pulsed source*_.

The concept relies on magnetic induction (Fig. 3). A magnetic core surrounds an evacuated accelerator tube where each core is enclosed within a conductive cavity. When a pulse is applied to a winding around the core, a longitudinal electric field is generated. By referencing one side of the pulsed source to the cavity interior, the electric field is net accelerating as $\oint E\cdot dl =0$ everywhere except in line segment *BC* (Fig. 3). This electric field is maintained for the pulse duration so long as the magnetic core does not saturate (i.e., the point at which leakage current around the core increases and the electric field collapses). This saturation effect is based on the core material, the cross-sectional area, and the volt-second product of the applied pulse^42^.

The LIA is charge to mass ratio independent, only requiring that the pulsed sources be synchronized, and the beam dynamics be properly managed. Experiments have demonstrated high dose-rate ion beams (2 A of He^+^, 2.4 ns pulse, and a 1 mm radius spot) using accelerator cavities originally designed for electrons^43,44^. Because the beam pipe radius can be arbitrary, accelerating multiple beamlets through a single accelerator cavity enables acceleration of multiple beams through a single accelerator^45,46^. Such an approach greatly simplifies a conformal FLASH-RT system. Since the beam pipe radius can be arbitrary, suppression of beam instabilities is straightforward in the LIA as the effect scales as the current divided by the beam pipe radius squared^38^.

To illustrate the extensive use of LIA technology, we list well known systems with energy > 5-MeV in Table 2^42,46–58^. These machines serve as the basis for numerous other uses that include intense charged particle beam propagation, free electron laser, dynamic FLASH radiography for weapons research, fusion experiments, and pulsed radiation effects studies.

**Table 2:** Worldwide linear induction accelerators (LIA) over 5 MeV.

Facility	Location	Energy (MeV)	Beam current (A)	Pulse Width (ns)	Instantaneous dose rate (Gy s^−1^, Ta @ 1 m)^a^	Maximum pulse repetition rate (Hz) [total no. pulses]	References
SLIA	Pulse Science Incorporated, USA	5.5	10,000	30	1.6 × 10^7^		^46^
Astron	Lawrence Livermore National Laboratory, USA	6	800	300	1.6 × 10^6^	1400 [100]	^47^
ETA-II	Lawrence Livermore National Laboratory, USA	6.5	3000	50	7.3 × 10^6^	5000 [50]	^48^
ETIGO-III	Nagaoka University, Japan	8	5000	30	2.1 × 10^7^	1	^49^
PIVAR	Centre d'études scientifiques et techniques d'Aquitaine-Cesta, France	8	3500	80	1.5 × 10^7^	1	^50^
SILUND-21	Joint Institute for Nuclear Research-Dubna, Russia	10	1000	60	7.6 × 10^6^		^51^
LIAXF/LIAXFU	Institute of Fluid Physics, China	12	2600	90	3.2 × 10^7^	1	^52^
FXR	Lawrence Livermore National Laboratory, USA	17	3000	60	9.3 × 10^7^	0.3	^53^
DARHT-II	Los Alamos National Laboratory, USA	17	2100	1600	6.5 × 10^7^	1	^54^
DARHT-I	Los Alamos National Laboratory, USA	20	2000	60	9.5 × 10^7^	1	^55^
AIRIX	Centre d'études scientifiques et techniques d'Aquitaine-Pontfaverger-Moronvilliers, France	20	4000	80	1.9 × 10^8^	1	^56^
DRAGON-I	Institute of Fluid Physics, China	20	3000	90	1.4 × 10^8^	1	^57^
LIA 30/250	Joint Institute for Nuclear Research-Dubna, Russia	30	250	500	3.5 × 10^7^	50	^42^
ATA	Lawrence Livermore National Laboratory, USA	45	10,000	75	4.1 × 10^9^	1000 [10]	^58^

^a^Based on: $\dot{D}$ = *1.7* × *10*^*4*^ ${I}_{Beam}$ ${Energy}^{2.65}$ (electrons onto Ta).

## Results

Photons generated by an LIA potentially provide broad area, deep penetrating, and high dose rate capability. To date, no MV broad beam photon sources have been characterized sufficiently to determine relevance to FLASH-RT. Here, we present experiments and measurements on the FLASH X-ray (FXR) accelerator used to accelerate electrons to 17 MeV and also on the Experimental Test Accelerator-II (ETA-II) with a nominal output energy of 6.5 MeV, but high repetition rate.

### Flash X-ray accelerator (FXR)

**Figure 4:**
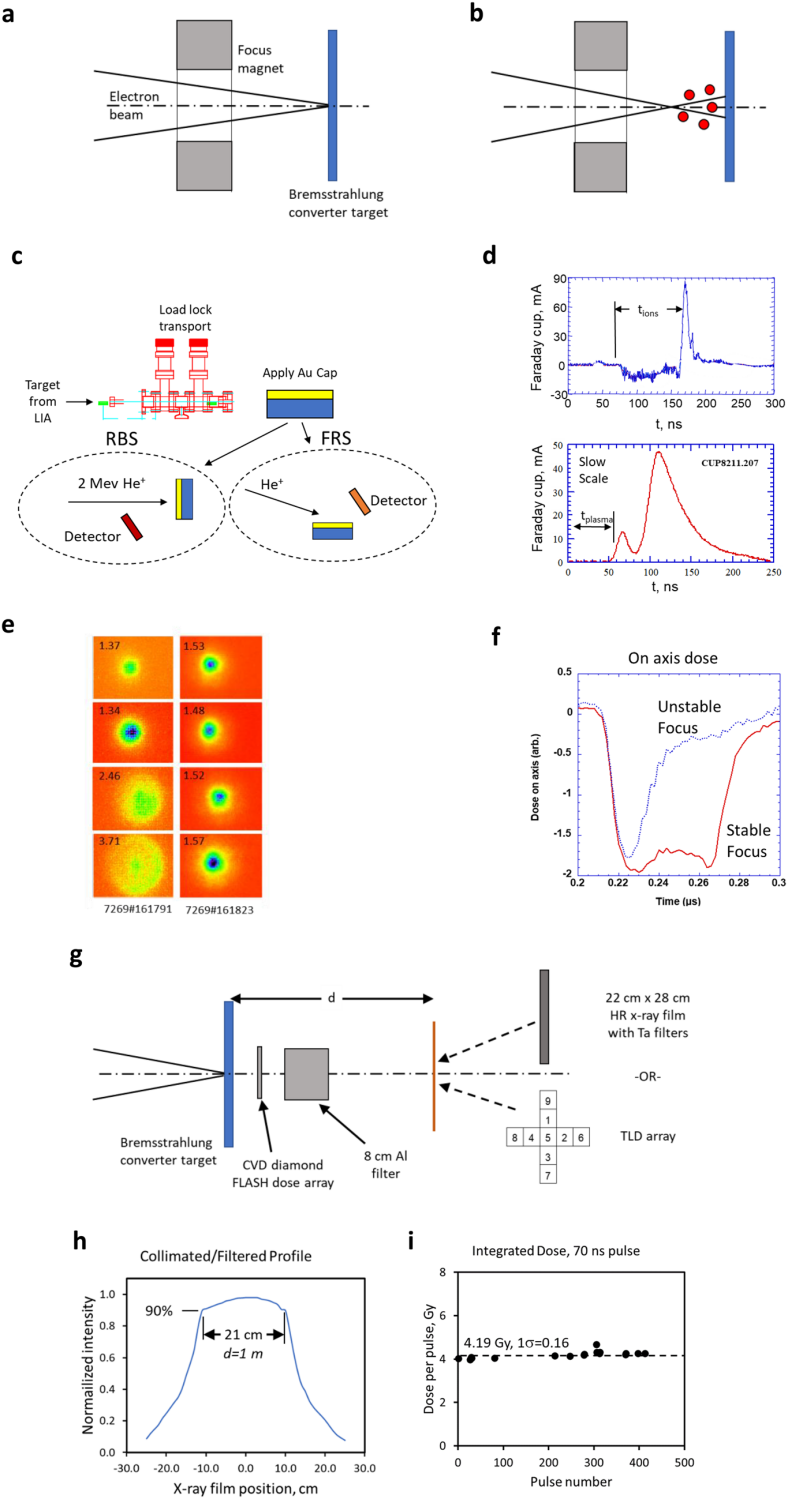
Experiments stabilizing the bremsstrahlung pulse in linear induction accelerators. (**a**,**b**) Depict a primary mechanism of spoiling the beam focus in a pulse; ions created by the electron beam interaction create a plasma where the space charge of the beam accelerates ions away from the target and partially neutralizes the beam and causes the waist to move upstream. (**c**) Depicts a method used to determine the surface and target contamination using Rutherford scattering and secondary ion mass spectroscopy. (**d**) Plasma measurements were made to determine the plasma expansion speed of both contaminants and target material. (**e**) Shows the dynamic bremsstrahlung spot behavior unmitigated (left) and mitigated (right) using focusing techniques. (**f**) Shows the impact of mitigating and not-mitigating back streaming plasma/ion from the target. (**g**–**i**) Shows the measurement geometry to determine bremsstrahlung field flatness and pulse-to-pulse repeatability.

FXR is an active research facility mainly used for hydrodynamic experiments^59^. Conversion to bremsstrahlung is with a Ta target. The beam is approximately 70 ns FWHM long with a focal spot that is nominally ≈ 1.5 mm to ensure high resolution imaging. For MV photon FLASH-RT to be viable using any pulse format, the focus of the beam must be stable pulse to pulse. It is well known that even in ultra-high vacuum monolayer formation on the target is rapid and scales in seconds as $\frac{3.2 \times {10}^{-6}}{P}$, where P is the pressure in mbar. Once the beam impacts the surface, this monolayer, as well as gas impurities present even in highly purified metals, is released and forms a plasma. Because the electron beam provides a strong negative potential, ions are extracted from the plasma boundary into the beam (Fig. 4a,b). The result is a dynamically varying focus on a prompt time scale.

In Fig. 4c, using a load lock transfer procedure, we performed analysis of the Ta bremsstrahlung converter targets operated at 10^–7^ to 10^–8^ T in the accelerator. Prior to analysis, an Au overcoating was used to confine and prevent contaminant introduction into the measurements. With Rutherford backscattering (RBS) at 2 MeV He^+^, forward Rutherford scattering (FRS), and secondary ion mass spectrometry (SIMS), we assayed the contamination as H_2_ @1.5 × 10^15^ cm^−2^ and C @3 × 10^15^ cm^−2^ on the surface with 6 ppm H_2_ in the Ta matrix.

To determine the density of the contaminant ions created during the beam pulse, we implemented high aspect ratio Faraday cups at various distances from the target (Fig. 4d). This high aspect ratio was used to ensure measurement errors due to secondaries generated from the ion impact were minimal. A negative bias (≈ − 50 V) stripped the low energy plasma electrons from the measurement. For a Faraday cup stand-off distance of 25 cm, we observed negative scattered electrons during the beam pulse and fast ions approximately 90 ns after the beam first impacts the target. It appeared that this fast ion pulse begins as the electron beam terminates. On the slow time scale, we observe two components: one at 50 μs and another at 80 μs. Each of these components correspond to an apparent speed of approximately 280 cm μs^−1^, 0.50 cm μs^−1^, and 0.31 cm μs^−1^, respectively which is in reasonable agreement with our hydrodynamic models^60^. From these signatures and the Faraday cup distance, we estimate the initial plasma density > 10^17^ cm^−3^ but falling off rapidly to approximately < 10^12^ cm^−3^ at about 500 ns.

While the initial plasma is too slow to immediately impact the FLASH beam, the ions are not. To determine the impact of the ions on the bremsstrahlung spot size, we used a multi-frame x-ray pinhole camera (see “Methods” section). The gate times were evenly spaced within a single bremsstrahlung FLASH pulse. What is clear from the first column of images (Fig. 4e) is a degradation of the focus in later frames. This effect manifests itself as a decreasing dose rate in time (Fig. 4f—marked “unstable focus”). This effect is far too fast to correct electronically (i.e., readjusting the focusing magnetic field). However, by using the pinhole camera for beam characterization and under focusing the beam (i.e., focused minimum beyond the target) we were able to achieve a net uniform spot and constant dose rate (Fig. 4f—marked “stable focus”). Typical optimized spot size was approximately 1.5 mm FWHM.

With an optimized focus, we performed total dose measurements during the 70 ns pulse to determine the collimated flat field and the shot-to-shot repeatability. The geometry for the measurement is shown in Fig. 4g. This particular measurement consisted of the bremsstrahlung converter target, a fast CVD diamond FLASH dose detector, an 8 cm thick low energy filter, and either thermoluminescent (TLD) or film detector at 1–2 m^61^. The CVD diamond FLASH detector is range thin to the photons so as not to perturb the measurement. Figure 4h shows a flat field for 90–100% as approximately 21 cm diameter at 1 m. This measurement corresponds to approximately a 350 cm^2^ area. We measured a 6° half angle through the collimator at 17 MeV electron energy. The cone angle was a result of the combination of the off normal electron trajectories and beam energy; the latter contribution scales as $\frac{1}{\gamma }$ where $\gamma =1+ \frac{{E}_{beam}}{{m}_{o}{c}^{2}}$, (*E*_*beam*_—incident beam energy, *m*_*o*_*c*^*2*^—energy of an electron at rest or 0.511 MeV).

To measure the pulse-to-pulse repeatability, we used the CVD diamond flash detector immediately after the target. This detector provides a fast signal that is recorded. For calibration we used an array of thermoluminescent dosimeters (TLDs). Bremsstrahlung was not generated on all pulses. In Fig. 4i, we measured the calibrated dose level and variation shot to shot. In that measurement, we observe approximately 4.19 Gy total integrated dose in the 70 ns pulse with a variation of approximately 1σ ≈ 0.16 corresponding to a 3.9% variation. This value corresponds to an instantaneous dose rate of approximately 6 × 10^7^ Gy s^−1^.

### The Experimental Test Accelerator (ETA-II)

**Figure 5:**
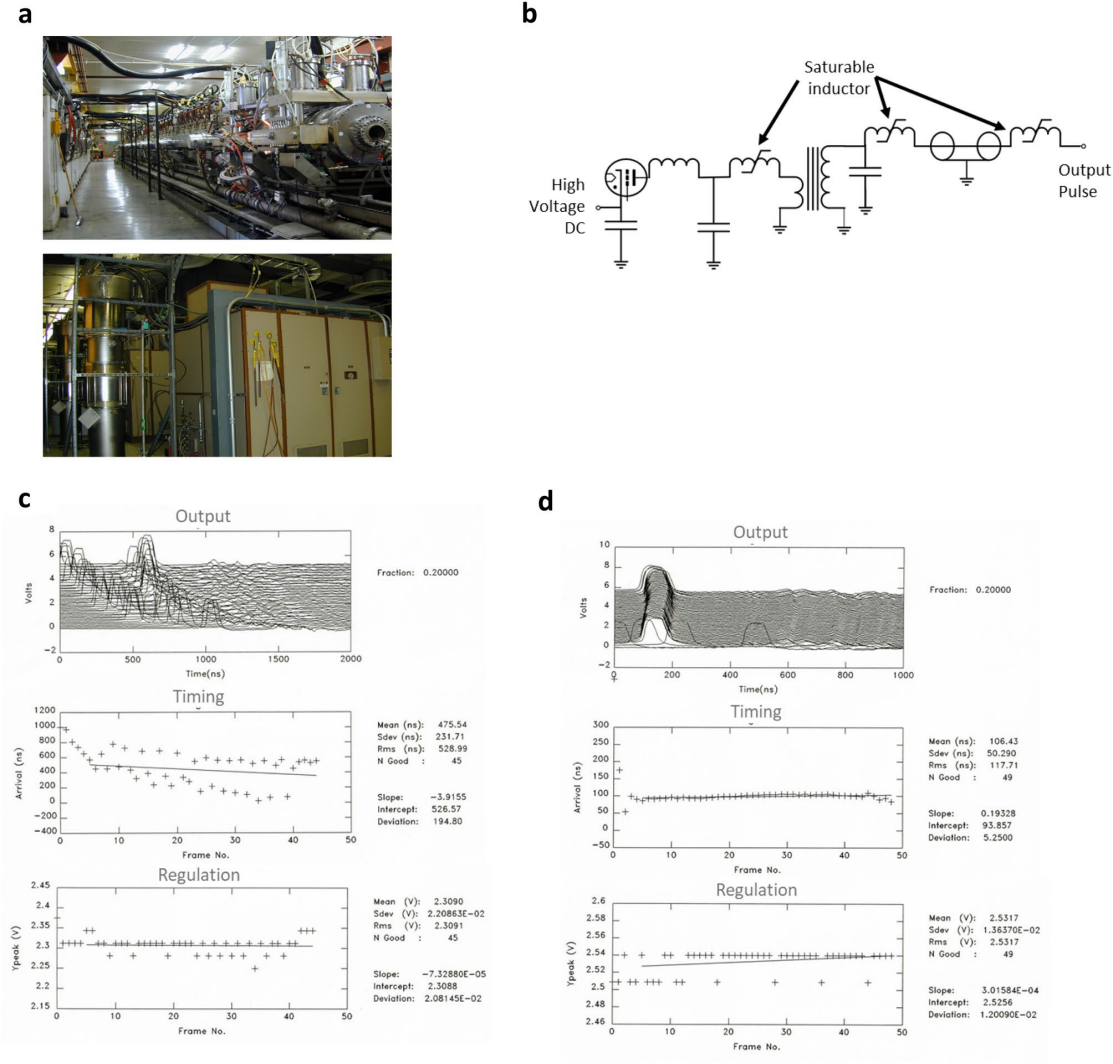

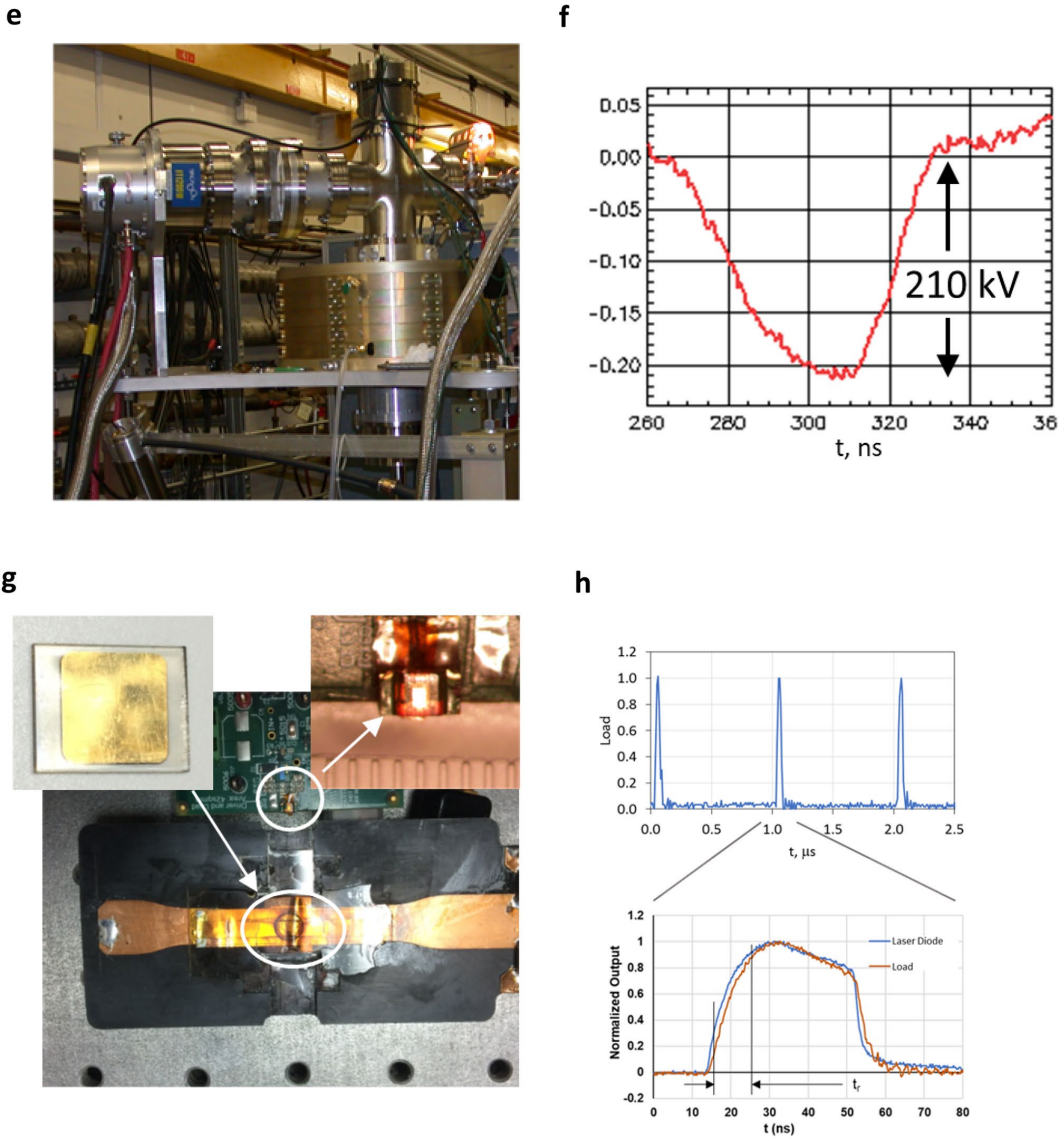
Development of high repetition rate LIAs. (**a**) The Experimental Test Accelerator-II (ETA-II) at Lawrence Livermore National Laboratory nominally rated at 3000-A beam current, 6.5 MeV, at 5 kHz (top) and the pulsed sources using thyratron gas switches and a Melville pulse compression transmission line (bottom). (**b**) Schematic of the pulsed source. (**c**,**d**) Effect of stabilizing the repeatability of the pulsed sources at 5000 Hz. (**e**,**f**) First tests on high gradient cavities operating at 42 kV per cell using SF_6_ insulation gas; we anticipate better than twice the performance using oil insulation. (**g**,**h**) Testing silicon carbide switching using the optical transconductance varistor (OTV) technology^64^.

The experimental test accelerator (ETA-II) is a high repetition rate system reconfigurable for accelerator development. It was designed to deliver highly stable electron beams with less than 1% energy variation, millimeter spot size, and submillimeter spot motion at 5000 Hz (Fig. 5a, upper). Initial use of the accelerator was in conjunction with a wiggler to generate electromagnetic energy similar to ESRF and IMBL (Table 1), but delivered 2 GW at 140 GHz mm-wave energy for fusion research studies^21,22,62^. Because of the pulse width requirement that was based on an older design, the machine gradient was only < 0.5 MV m^−1^.

Here, we explore repetition rate operation of an LIA. The pulsed source for ETA-II utilized thyratrons (Fig. 5a, large cabinets) driving a Melville line (large vertical cylindrical structure to the left)^63^. The overall schematic is shown in Fig. 5b. A capacitor is charged and switched into the non-linear Melville transmission line. This pulse is sent through a step-up transformer and series saturable inductors that successively compress the energy in time to provide high peak power for each LIA core. At full voltage operation, the pulsed sources deliver 135-kV into a 2-Ω load at 5000-Hz at better than 90% efficiency. Four pulsed sources were used to drive the accelerator; three drive sixty accelerator cavities and one drives the injector to deliver nominally ≈ 3000-A electron beam at 6.5-MeV in a 70-ns pulse.

Stability of the system (pulse-to-pulse variation and output energy) is determined by the state of the magnetic cores both in the accelerator and also in the pulsed sources. Excessive internal reflected energy improperly returned the magnetics to a varying state (i.e., reset). We observed the system to exhibit bipolar state behavior with large variations in timing. The upper plot (Fig. 5c) shows a multipulse burst from the pulsed source at 5000 Hz where the z axis is pulse number. The timing variations in this data set were so large that five pulses of the burst were outside the 2000 ns capture window. The data summary plot for timing (middle frame) and pulse-to-pulse energy regulation (lower frame) are also shown in the figure. While the timing variation was large, the pulse-to-pulse regulation was 1σ < 1%. To achieve lower jitter, we reduced reflected energy by better matching of the Melville line to the accelerator and also used more aggressive damping in the core reset system. The final result is shown in Fig. 5d. Although a slight timing variation was seen for the first two pulses, the remaining pulses showed a timing variation of < 5 ns while showing a pulse-to-pulse energy regulation of ≈ 0.5%. This result was confirmed with a magnetic energy analyzer at the end of the accelerator. If converted to bremsstrahlung, the net dose rate variation would be ± 0.71%.

We are presently designing and testing accelerator cavities with a goal to achieve higher acceleration gradients. The experimental arrangement is shown in Fig. 5e. These particular cavities were initially insulated with SF_6_ pressurized gas which limited breakdown performance. At this stage of testing, we were able to achieve 0.210 MV across 0.165 m (Fig. 5f) or ≈ 1.3 MV m^−1^ for a pulse width of 40 ns FWHM. This gradient is approximately 2–3 times the gradient of ETA-II and with oil insulation, we expect to achieve another doubling of that gradient.

To replace the existing pulsed source, we are testing high repetition rate, single crystal, semi-insulating SiC photoswitches driven by pulsed laser diodes (Fig. 5g–h)^64^. We have also tested and utilized a configuration that charges capacitors in parallel and selectively discharges them in series to allow output voltage variability, and hence variable control of the electron final energy over a wide range^65^. Figure 5g shows a device under test in a low inductance geometry. In this particular geometry, the SiC (upper left inset) was integrated into a polyimide epoxy structure. This particular structure has been tested to approximately 30 kV under oil. Contacts were evaporated gold. The diode laser and driver are shown in the larger photo; the surface mount laser diode is shown in the upper right inset. Scale is 1 mm spacings. This particular diode was rated for 70 W pulsed output in the IR spectrum with a 0.1% duty cycle. Figure 5h shows the output at 1 MHz pulse repetition frequency. Risetime was approximately 12 ns and was limited by the diode driver circuit.

## Discussion

**Figure 6:**
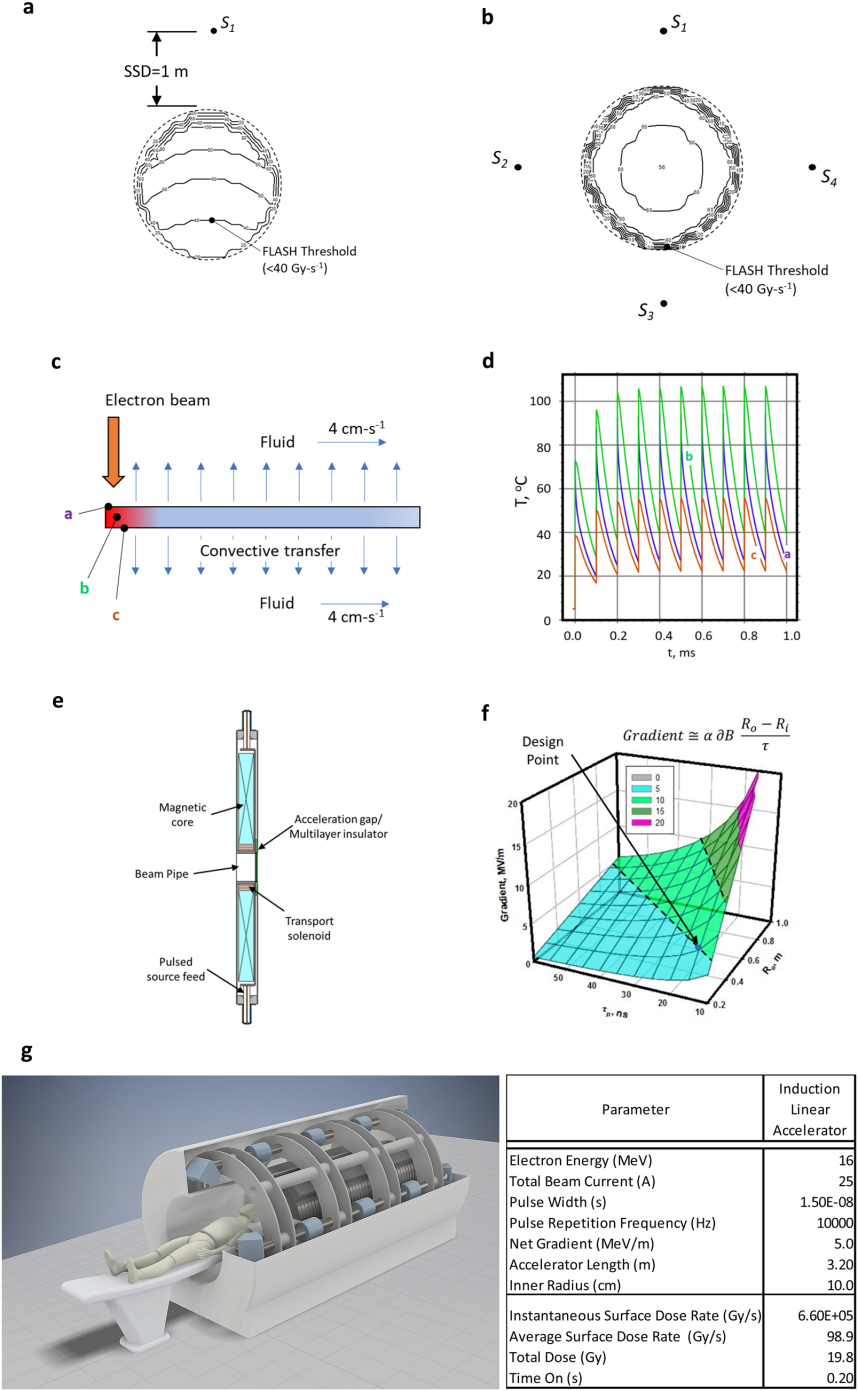
Point design considerations for a high gradient linear induction accelerator for FLASH-RT. (**a**,**b**) 100 Gy s^−1^ single sources and four 25 Gy s^−1^ multisource dose rate calculations to deliver above the tissue sparing threshold and reduced toxicity. Single source results in a below tissue sparing dose at the exit edge whereas the four sources provide a nearly uniform distribution above the 40 Gy s^−1^ heathy tissue sparing threshold. (**c**,**d**) Cooled multilayer target considerations. Multilayer target enables rapid cooling from the target interior causing the target temperature to equilibrate at approximately 110 °C. (**e**,**f**) Induction cavity design based on volt-second properties of the core and the dimensions, where: α—cavity packing efficiency, ∂B—flux swing of the magnetic core, R_i_—core inner radius, R_o_—core outer radius, and τ—accelerator pulse width. For our design point, we take R_i_ = 0.1-m_,_ ∂B ≈ 0.6 T (NiZn ferrite), and α  = 60% 73 44. (**g**) Concept FLASH RT system using a linear induction accelerator (LIA) providing four lines-of-sight. LIA is on axis with the patient. Blue elements are magnetic focusing elements that direct the electron beam to the patient. Accelerator is 3.2 m, overall system length is 3.5 m less the patient couch.

The MRT results using a synchrotron at 95 keV suggests that both the periphery and exit dose rate need to be above the healthy tissue sparing threshold to minimize toxicity^29^. Based on this conclusion, we developed an approach for a FLASH-RT system using an LIA (Fig. 6) by first calculating the dose rate distribution for two cases. The first case was a single source at 100 Gy s^−1^ at 1 m (Fig. 6a). The second case was four separate sources placed symmetrically around the volume, each at 25 Gy s^−1^ at 1 m (Fig. 6b) or 100 Gy s^−1^ total. Each case relied on percent depth dose (PDD) experimental curves^66,67^. For simplicity, we used an idealized cylindrical volume with the mean value of the human abdominal circumference of ≈ 1 m or radius of ≈ 16 cm^68^. Source surface distance (SSD) was 1 m for all sources. Electron energy was 16 MeV. Conversion to bremsstrahlung was based on a Ta target.

In Fig. 6a, while a heathy tissue sparing dose rate > 40 Gy s^−1^ is delivered nearest the single source, about 25% of the volume is below that dose rate. In Fig. 6b, a symmetric configuration of four sources achieved 50% beyond the required healthy tissue sparing dose rate, or about 60 Gy s^−1^ throughout the majority of the volume. The minimum dose rate in that volume decreases slightly to about 56 Gy s^−1^ in the center of the volume. While dose build up was considered using the deposition curves, dose rate at the volume edge increased rapidly to 40 Gy s^−1^ in 1–2 cm because of the added contribution from the other sources. This result suggests that for MV bremsstrahlung FLASH-RT, multibeam delivery is necessary to remain above the healthy tissue sparing threshold. Thus, we baseline our approach using four sources, but to minimize the possibility of loss of a source, resulting in below the healthy tissue sparing dose rate, we rely on a single accelerator generating all of the separate beamlets.

We have demonstrated conversion of the electron beam to bremsstrahlung using Ta on both FXR and ETA-II and presented measurements of the physical processes of the beam-target interaction to produce a predictable through collimator dose. The measurement showed a uniform field of ≈ 350 cm^2^ at 1 m with a dose ≈ 4.2 Gy per pulse integrated over 70 ns. Instantaneous dose rate was ≈ 6 × 10^7^ Gy s^−1^. The pulse-to-pulse variation was 1σ ≈ 0.16 or 3.9%. This result is in contrast with the largest FLASH-RT field to date of a 55 cm^2^ field using electrons^17^.

On ETA-II, we demonstrated stable operation at 5 kHz both in timing (< 5 ns) and energy variation delivered by the pulsed source (< 0.5%).

Using photonic switches, we demonstrated 1 MHz repetition rates. Using the controllability of the device, the pulsed sources can be simplified to provide electron energy variability.

Here we use a 1.5 mm spot diameter and four separate beamlets. Added beamlets reduce the bremsstrahlung converter requirement for a given dose rate and result in a healthy tissue sparing level. For scaling, we use the well-known relationship of electrons to bremsstrahlung dose rate as: $1.7 \times {10}^{4} I{V}^{2.65}$ (in Gy s^−1^ for Ta at 1 m distance), where *V* is in MV and beam current *I*, is in kA.

The pulsed beams delivered for our ETA-II measurement had an instantaneous power density on the Ta target ≈ 10^12^–10^13^ W cm^−3^. At these power densities, the target undergoes rapid hydrodynamic expansion on the microsecond time scale and is therefore single use. FLASH-RT requires an instantaneous dose-rate > 1.8 × 10^5^ Gy s^−1^^14,15^. These lower rates allow using electron beam current and repetition rate as a free parameter for the target approach.

Short duty cycle (≈ 30 s) DC x-ray tubes using thermal inertia have achieved almost 200 kW-cm^−2^^69^. 25 Gy s^−1^ total requires a 6.25 A beam at a 10 kHz rate with 15 ns pulses on each of the converter targets (“Methods” section). For a 1.5 mm spot size, the instantaneous power flux is 850 kW cm^−2^ which is still clearly outside the range of present state-of the-art.

Two target approaches are still possible: Heat generated from a 1.5 mm electron spot size at 16 MeV and 6.25 A can be dissipated by rotating the target at an equivalent linear speed of 15 m s^−1^. For an 8 cm radius disk, approximately 6 rotations are required for an 0.2 s treatment cycle of 20 Gy. Net local temperature rise is 725 °C and well within the Ta melting temperature of 3017 °C. For simplification, as a second approach, we modeled a liquid cooled multilayer target where each layer is designed to absorb ≈ 4% of the electron beam energy (total of 25 layers)^70^. In this model, each layer is allowed to convect heat away into a moving fluid (Fig. 6c,d). For the fluid, we assumed a convective heat transfer coefficient of 100 W m^−2^ K^−1^, a flow rate of 4.2 cm s^−1^ and allowed a 50 °C temperature rise. Peak equilibrium temperature is approximately 110 °C.

The high gradient LIA cavity concept is shown in Fig. 6e, with the gradient given by: $\alpha \partial B\frac{{R}_{o}-{R}_{i}}{\tau }$, where *α* is the packing efficiency, *∂B* is the flux swing of the cores, *R*_*o*_ and *R*_*i*_ are the magnetic core outer and inner diameters, respectively, and *τ* is pulse width^71^. To provide the insulator interface between the magnetic core and vacuum region for the electron beam, we use multilayer insulators. These structures are made up of periodic layers of conductive and insulating material laminated into a monolithic structure. The structure provides an increase in breakdown electric field of up to 4×, is insensitive to polarity effects, photon and charge particle flux, and suppresses beam breakup instability resonances^72–75^.

To allow enough room for multiple beams while keeping the cavity small to avoid unwanted beam breakup modes, we select a ferrite inner radius of *R*_*i*_ = 0.1-m. We also take_,_ ∂B ≈ 0.6 T (NiZn ferrite), and α = 60% because of the insulator configuration^42^. The surface for the optimization trading *τ* and *R*_*i*_ to achieve a given gradient is shown in Fig. 6f. We show a design point of 5 MV/m at a 15 ns pulse width for *R*_*o*_ ≈ 0.3 m.

A single LIA is used to accelerate separate beamlets in an approximately 14 cm diameter beam pipe. While four beams are shown, eight or more can be easily implemented. Beam transport is managed through the accelerator with solenoid coils and integrated steering similar to FXR^59^. The added steering capability enables generating of oblique rays to potentially allow a closer approximation of multibeam conformal therapy. At 16 MeV, the system would be approximately 3.2 m long (Fig. 6g) delivering an average dose rate of 60 Gy s^−1^ (≈ 4 x 10^5^ Gy s^−1^ instantaneous) through an idealized 16 cm radius volume, but more uniformly distributed.

## Methods

We developed Fig. 2 and Table 1 by performing an exhaustive search of FLASH-RT systems in the open literature leveraging the work of Vozenin and Bourhis and estimated the instantaneous dose rate where possible^14,15^. We also use the work of Esplen, Wilson, and Darafsheh^5,6,8^ but in an attempt to standardize methodology, we used spot size, range at energy, and average beam current to determine dose rates. When possible, we relied on the text of the original papers and cross checked with our estimates. For electron range, we used stopping power data from El-Ghosssain over four tissue types^76^. For bremsstrahlung photons, we used the PDD data by Hill for low energy and those by Narayanasamy and Feye for MV energy^66,67,77^. The intense photon sources at ESRF and IMBL use wigglers. The spectra provided were bremsstrahlung-like to first order and sufficient for this parameter survey; no added corrections were made. Turner provided the correction between a water phantom and tissue; again, no changes were necessary for our estimates^78^.

For our dose-rate calculations, we used one range depth (i.e., I/I_o_ ≈ e^−1^) for electrons and photons. For protons, where the data was provided, we used the 10% point of the distal edge enclosing the Bragg peak at energy using the dose depth curves from Kang for the energy 28–227 MeV^79^. As those data were taken with water, we scaled to range data for tissue by Usta averaging over eight tissue types^80^.

The x-ray pinhole camera for FXR was designed and built for measuring the x-ray spot size as a function of time. It consisted of a “pinhole” composed of a series of tungsten cylinders stacked in a 15 cm long stainless-steel holder. Each cylinder had a hole formed by EDM, ranging in diameter from 200 to 760 μm. The cylinders were stacked to approximate a double-tapered cone, with a section of 200 μm pinholes by 4 cm long in the center. Behind the single pinhole, the camera had four gated channels that record the x-ray images to CCD cameras. Each of the channels had a scintillator array to convert the x-rays to visible light, a mirror and relay lens to image the scintillator onto the input of a microchannel image intensifier, and a CCD camera fiber-optically coupled to the output of the intensifier. The unit was shielded from external radiation using W powder packed between aluminum plates. Each camera had a frame buffer that stores the image for readout after each shot. The cameras had standard analog video output, but it was transmitted to the FXR control room via a 4-channel fiber-optic link. Arming of the camera frame buffers was also done through this fiber optic link. The video was digitized by a Scion frame-grabber and computer. NIH Image J was used for local analysis of the images. Calibration was done with geometric considerations and cross correlating the results with other spot size measuring techniques such a observing the blur over an opaque edge.

Simplified dose rate distributions were developed using experimental PDD curves at the proper energy. This approach takes the dose build up zone into account as well as the bremsstrahlung spectrum. The calculations were done in 2D using standard ray tracing techniques to each ≈ cm sized voxel. The array was then processed to provide the dose map. In addition, we used numerical approximations to crosscheck our results. Namely we use: ${I}_{T}= \sum_{i=1}^{N}{I}_{i}$, where ${I}_{T}$ is the total dose rate and ${I}_{i}$ is each source rate *i*. In the dose fall off region, we estimate the dose rate as a function of depth using an exponential function; the PDD curves correlated well to this function, with ${\chi }^{2}=0.9997$.

The key requirements for managing radiation induced toxicity is to maintain > 40 Gy s^−1^ or preferably > 100 Gy s^−1^. While it is not completely clear what the instantaneous dose rate should be, some suggest ≈ 10^6^ Gy s^−1^^5^. Our goal is high gradient requiring that the pulse width of the LIA be kept as short as possible but is limited by magnetic core response. Typical response is as fast as 6–8 ns^81^. This result allows a practical minimum pulse width of approximately 15 ns. As stated earlier, our calculation is based on electrons to bremsstrahlung instantaneous dose rate scaling as: $1.7 \times {10}^{4} I{V}^{2.65}$, where *V* is in MV and beam current *I*, is in kA and is a relatively good predictor for dose rate in Gy s^−1^ for Ta at 1 m distance. Instantaneous dose rate is thus defined by the current and electron energy and average dose rate is determined by the pulse repetition rate and pulse width.

### Disclaimer

Reference herein to any specific commercial products, process, or service by trade name, trademark, manufacturer, or otherwise, does not necessarily constitute or imply its endorsement, recommendation, or favoring by the U.S. government or Lawrence Livermore National Security, LLC. The views and opinions of authors expressed herein do not necessarily state or reflect those of the U.S. government or Lawrence Livermore National Security, LLC, and shall not be used for advertising or product endorsement purposes.
